# Development and Validity of a Questionnaire on Dyslipidemia Dietary Knowledge

**DOI:** 10.1155/2019/5382130

**Published:** 2019-06-02

**Authors:** Na Liang, Qiuli Zhao, Yuhua He, Jingshu Li, Li Yang

**Affiliations:** ^1^Tourism & Health Vocational College of Zhejiang Zhoushan Archipelago New Area, 99 Xueyuan Road, Zhoushan, Zhejiang Province 316000, China; ^2^School of Nursing, Harbin Medical University, 246 Xuefu Road, Harbin, Heilongjiang Province 150086, China; ^3^Department of Gastrointestinal Surgery, West China Hospital, Sichuan University, No.37 Guo Xue Xiang, Chengdu, Sichuan Province 610041, China; ^4^The 2nd Affiliated Hospital of Harbin Medical University, Harbin Medical University, 148 Baojian Road, Harbin, Heilongjiang Province 150086, China; ^5^School of Nursing, Qingdao University, 38 Dengzhou Road, Qingdao, Shandong province 266021, China

## Abstract

**Objective:**

Dyslipidemia is one of the most important modifiable risk factors for cardiovascular disease. Diet and lifestyle interventions positively contribute to the management of dyslipidemia. Adequate knowledge of the correct diet for dyslipidemia is an effective preventive strategy against cardiovascular disease.

**Method:**

This study developed a questionnaire to test dyslipidemia dietary knowledge. The initial, multiple-choice dyslipidemia dietary knowledge questionnaire (DDKQ) was formed based on a literature review of previous studies. Two Delphi rounds were performed to ensure content validity and face validity, items were pretested and filtered through item analysis, and the reliability and validity of the final questionnaire were checked.

**Results:**

The DDKQ consisted of 5 parts, with 25 items in total. It demonstrated adequate content validity (0.94), face validity, internal consistency (KR20 = 0.67), and test-retest reliability (0.74). The mean item difficulty (P) was 0.55 and ranged from 0.15 to 0.83, and the mean item discrimination index (D) was 0.36, ranging from 0.21 to 0.58. The questionnaire was also able to distinguish between participants with/without a medical background; medical workers produced significantly higher total scores (16.70±2.84 vs. 14.57±4.26, p< 0.05).

**Conclusion:**

The DDKQ is a reliable and valid measure of dyslipidemia dietary knowledge. It is suitable for providing scientific assessments for targeted health education interventions.

## 1. Introduction

As a modifiable and independent risk factor, dyslipidemia plays a causal role in the pathogenesis of cardiovascular disease (CVD), which now is the main cause of death in the world [1]. Although CVD deaths are decreasing in the developed world, they continue to rise in developing countries [2]. Forecasts suggest that in China, as the population increases and becomes generally older, the incidence of CVD will increase by over 50% in the following 20 years [3].

As an extensive term, dyslipidemia refers to a range of lipid disorders. Dyslipidemias can be generally divided into low high-density lipoprotein cholesterol (HDL-C), elevated low-density lipoprotein cholesterol (LDL-C), atherogenic dyslipidemia, hypertriglyceridemia, mixed lipid disorders, and excess lipoprotein(a) [4]. The majority of lipid disorders (80%) are associated with lifestyles and diet, despite the significant role of familial disorders (20%) [5].

As per recommendations, the primary therapy for hypercholesterolemia specifies lifestyle and dietary modifications [6]. Several research studies have shown the efficacy of lifestyle interventions and dietary changes in decreasing LDL-C and cardiovascular issues [7, 8]. Dietary changes include those that limit total cholesterol, fat, saturated fat, and energy, with the inclusion of two grams of plant stanols/sterols every day and enhanced consumption of soluble fiber [9, 10]. These dietary changes can decrease LDL-C, triglycerides, body weight, and so forth. All adults should be encouraged to adopt a healthy diet. However, these factors are most significant for people with enhanced LDL-C levels [11].

Researchers have developed a variety of evaluation tools for testing the effect of interventions. These consist of (1) tools for assessing the nutrient composition of a diet, such as the food frequency questionnaire (FFQ), diet history, 24-hour recalls, and dietary records; (2) assessing dietary patterns, such as via the Food Screener [12], and Fat Intake Scale [13], which assess dietary patterns to some degree and/or fat intake, but ignore the role of other macronutrients in the pathogenesis of hyperlipidemia; (3) dietary intake behavioral tendency questionnaires, such as Dietary Behavior Questionnaire [14]; and (4) cognitive assessment tools that address behavioral attitudes, beliefs, and self-efficacy, such as the questionnaire of Huang et al., which assesses knowledge, attitudes, and practice regarding serum lipids [15], and the Cholesterol-Lowering Diet Self-Efficacy Scale [16]. Only Huang et al.'s questionnaire involved the assessment of lipid knowledge (such as the risk of dyslipidemia and the treatment of dyslipidemia); there exist no specific tools to test dyslipidemia dietary knowledge. Extensive nutritional knowledge and behavioral changes can lead to significantly reduced LDL-C levels [17]. Knowledge is one of the components that can drive changed food habits [18, 19]. Therefore, to prevent the occurrence of dyslipidemia, it is important to strengthen knowledge of dyslipidemia diets.

The research objectives were to (1) develop a dyslipidemia dietary knowledge questionnaire (DDKQ) that represents a scientifically-based and reliable evaluation tool for conducting investigations and interventions in the future and (2) assess the psychometric properties of the DDKQ. The DDKQ is the first scale developed for Chinese adults.

## 2. Methods

This study was conducted according to the guidelines laid down in the Declaration of Helsinki and all procedures involving human subjects/patients were approved by the 2nd Affiliated Hospital of Harbin Medical University, China.

### 2.1. Participants

Participants were recruited from the 2nd Affiliated Hospital of Harbin Medical University and Medical Center and local community groups in Harbin City, China. All participants signed the informed consent form, were provided with a verbal explanation about the purpose of the study, and informed that participation was voluntary, confidential, and anonymous. The criteria for inclusion were that the participants were volunteers of 18 years of age or older, who are able to read and respond to questionnaires. Participants were excluded if they had any of the following characteristics: (1) dementia, or severe psychiatric disorders, (2) altered consciousness, (3) deafness or blindness. Some participants were diagnosed with dyslipidemia, according to laboratory indicators (TC≥6.22mmol/L; LDL-C≥4.14 mmol/L; HDL-C<1.04 mmol/L; TG≥2.26mmol/L); the diagnostic criteria are based on guidelines on prevention and treatment of dyslipidemia in Chinese adults.

### 2.2. Procedure

**Figure 1:**
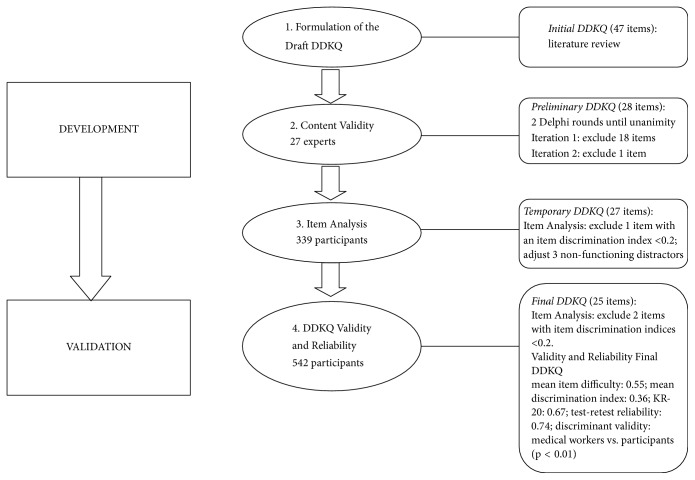
Schematic representation of the Dyslipidemia Dietary Knowledge Questionnaire (DDKQ) development and validation process.

The study consisted of two phases: (1) instrument development and (2) evaluation of psychometric properties. The process was divided into four steps (Figure 1).

**Step 1 (formulation of the draft DDKQ).**

DDKQ was formulated in accordance with the Guidelines on Prevention and Treatment of Dyslipidemia in Chinese Adults, also developed from literatures of previous studies, monographs on dyslipidemia dietary knowledge and the authors' clinical experience. Six components were assessed: fat knowledge, cholesterol knowledge, dietary fiber knowledge, phytosterol knowledge, total energy knowledge, and healthy lifestyle knowledge. Initially, 47 multiple-choice questions were constructed with four response alternatives per question: one correct answer and three distractors. Items with correct responses were scored as 1 point, while incorrect responses were scored as 0 points.

**Step 2 (content validity).**

The preliminary questionnaire's content was validated by performing two Delphi rounds, in which the concepts were presented to a panel of 27 experts. The construction of the expert panel encompassed most relevant departments, namely, clinical nutrition, cardiology, endocrinology, geriatrics, and professional teachers. The panel consisted of 7 clinical nutritionists, 7 university nutrition professional teachers, 6 related clinical doctors, and 7 related clinical nurses. The experts were selected according to whether they satisfied the following criteria: (1) had a profound interest and rich clinical knowledge of nutrition, especially with respect to lipids; (2) had been engaged in related work for more than 10 years, or had at least a master's degree. In addition, the experts were distributed among different units and did not know each other's names or departments, to avoid communication between them.

Experts were invited to review the items for content, breadth, applicability, and to rate each item in terms of its validity and relevance. Following the first Delphi round, the expert opinions and suggestions were summarized, and the DDKQ was adjusted according to them. After the second Delphi round, the content validity index (CVI) of each item was calculated. Any item with a CVI greater than 0.78 was considered excellent, whereas the goal for Scale-Content Validity Index/Average (S-CVI/Ave) was 0.90 or higher [20]. To test face validity, experts were asked if all questions were clearly worded and unambiguous.

**Step 3 (item analysis).**

Items in the preliminary questionnaire that satisfied all of the following conditions were modified to formulate a temporary questionnaire: (1) item difficulty more than 0.9 or less than0.1 [21]; (2) discrimination index less than 0.20 [22]; (3) contained a non-functioning distractor [23].

**Step 4 (DDKQ reliability and validity).**

To characterize the temporary DDKQ, item analysis was repeated until the final DDKQ was formed. Then, the reliability and validity of the final DDKQ were tested.

## 3. Data Analysis

### 3.1. Characteristics of the Sample

The clinical-demographic characteristics of the experts and participants were assessed by descriptive analysis (including averages and standard deviations).

### 3.2. Content Validity Index

The expert panel was asked to evaluate whether each item was relevant for measuring dyslipidemia dietary knowledge, using a four-point Likert scale (1 = not relevant, 2 = a bit relevant, 3 = relevant, 4 =highly relevant). The I-CVI was calculated as the number of experts who gave a rating of 3 or 4, divided by the total number of experts. The S-CVI/Ave was calculated by averaging the I-CVIs.

### 3.3. Item Analysis

Item analysis is a valuable, yet relatively simple, procedure performed after test development that provides information regarding the reliability and validity of test items [24]. It also characterizes the difficulty of questions (the difficulty index), and whether the questions were able to discriminate between participants who performed well on the test and those who did not (the discrimination index). Analysis of distractors is another important part of item analysis. It provides information regarding the individual distractors and the key of a test item. Using these tools, the researcher is able to modify or remove specific items when formulating subsequent tests [23].

The difficulty index value of an item is defined as the proportion of respondents who answer the question correctly [25]. Possible values range from 0.0 to 1.0. Items with a difficulty (P) greater than 0.9 were considered “too easy” and were deleted. Similarly, items with a difficulty less than 0.1 were considered “too difficult” and were also excluded [21].

The discrimination index (D) describes the ability of an item to distinguish between high and low scorers. To calculate the discriminative value of each item, the respondents were divided into the 27% who scored highest and the 27% who scored lowest. Then, the following formula was used:

(1) $$\begin{array}{c} \frac{number\;\;of\;\;correct\;\;answers\;\;in\text{ “}high\text{” }group-number\;\;of\;\;correct\;\;answers\;\;in\text{ “}low\text{” }group}{total\;\;number\;\;in\;\;both\;\;groups} \end{array}$$

A value above 0.39 indicated excellent discrimination, 0.30–0.39 was considered good, 0.20–0.29 suggested the item needed to be checked, and less than 0.20 denoted an item with low discriminatory power that should be replaced [26].

An item (multiple-choice question) [MCQ] contained a stem and four options, consisting of one correct option (key) and three incorrect (distractor) alternatives. Any distractor that was selected by less than 5% of participants was considered a non-functioning distractor (NFD) [23] and otherwise a functioning distractor. Ideally, low-achieving participants who have not mastered the subject should choose the distractors more often, whereas high scorers should discard them more frequently in the process of choosing the correct option. By analyzing the distractors, it is possible to identify errors and items with NFDs and remove them from future assessments [27].

### 3.4. Internal Consistency

The Kuder-Richardson Formula 20 (KR-20) was used to calculate the internal consistency reliability coefficient for items with dichotomous choices (e.g., correct/incorrect). A value of 0.70 or greater is generally considered acceptable [28].

### 3.5. Between-Group Differences

Differences between groups were identified using the Mann-Whitney U-test. Statistical significance was defined as* p*<0.05 (two-tailed).

### 3.6. Questionnaire Stability

To verify the stability of the questionnaire, a retest took place after approximately two weeks. Pearson's correlation was used to measure the test-retest reliability. It has been suggested that Pearson's correlation between replications of a test should be at least 0.7 [29, 30].

## 4. Results

### 4.1. Demographic Characteristics of Participants

Twenty-seven experts took part in the expert validation. Three experts were excluded from the second Delphi rounds because they were unavailable due to business travel. Twenty of the 27 experts were 45 years of age or younger. Nine of the 27 experts had 5–10 years of relevant experience, 9 had 10–15 years of experience, and 9 had more than 16 years of experience. Ten of the 27 experts had intermediate seniority, 10 had vice-senior titles, and 7 had senior titles. Seven of the 27 experts had bachelor's degrees, 7 had master's degrees, and 13 had doctoral degrees.

In Step 3 360 participants were surveyed, of whom 21 were excluded (12 refused to take part and 9 provided incomplete information), leaving 339 (94.17%) participants (156 males, 183 females). The mean age of the participants was 47.71 years (*SD* = 16.62 years, range 18–95 years). The distribution of ages was 143 (18–44 years), 102 (45–59 years), and 94 (≥60 years). Dyslipidemia was present in 149 individuals, whereas the remaining 190 acted as healthy adult controls. The 339 participants' demographic data are presented in Table 1.

Three months after item analysis (Step 3), a total of 600 participants were recruited for the survey in Step 4. Of these, 58 were excluded due to refusal to participate (n =31), provision of incomplete information (n =24), or other reasons (n = 3), leaving 542 (90.3%) participants who were included in the internal reliability and validity assessments (249 males, 293 females; mean age = 50.49 years,* SD* = 16.42 years, range 18–89 years). The distribution of ages were 189 (18–44 years), 176 (45–59 years), and 177 (≥60 years). Dyslipidemia was present in 268, and the remaining 274 were healthy adults. Most participants (n= 496) had no medical background, except for 46 who were medical workers. The remaining 542 participants' demographic data are presented in Table 2.

### 4.2. Content Validity

Through the Delphi expert enquiry method, data on the questionnaire content validity were obtained. Following the first Delphi round, the experts suggested reducing/removing items that pertained to phytosterol because absorption is limited and the effect is weak. Accordingly, 18 items were deleted. In the second Delphi round, expert opinions were relatively uniform. The content validity index (CVI) of each item was calculated. Only one item did not achieve the criterion of I-CVI<0.78 and was deleted. There were 28 remaining items. The S-CVI/Ave of the final DDKQ was 0.94. The content validity scores of each item are shown in Table 3.

### 4.3. Item Analysis

In Step 3, with in which 339 participants' data were analyzed, 4 items were identified with a discrimination index less than 0.2. This, in combination with the opinions of the team, led to 1 item being deleted and 3 preserved for subsequent verification. There were 3 non-functioning distractors in 3 items, which were modified. In Step 4, analysis of the 542 participants' data revealed 2 items with a discrimination index less than 0.2, which were deleted. After the modification, there were non-functional distractors in the final DDKQ. The final DDKQ consisted of 25 items and 5 parts. The mean item difficulty (P) was 0.55 (range: 0.15–0.83) and the mean item discrimination index (D) was 0.36 (range: 0.21–0.58). Item difficulty and the discrimination index values are shown in Table 3.

### 4.4. Internal Consistency Reliability and Test-Retest Reliability

The DDKQ's internal consistency, as measured by KR-20 was 0.67. Sixty participants completed the questionnaire twice, with approximately 2 weeks between the first and second administration of the questionnaire, to evaluate test-retest reliability. The overall reliability was acceptable (*r* = 0.74,* p*<0.001).

### 4.5. Discriminant Validity

Of the participants, 46 were medical workers. Therefore, 46 non-medical worker participants were selected randomly, and the two groups compared. There was a statistically significant difference between the scores of medical workers and participants with no medical background (16.70±2.84 vs. 14.57±4.26,* p*< 0.05). As expected, participants without medical experience scored lower than medical workers, indicating that the questionnaire has good discriminant validity.

## 5. Discussion

This is the first DDKQ designed for, and validated in, Chinese adults. The aim of this study was to develop a reliable and valid questionnaire covering all aspects of diet knowledge about dyslipidemia, which could be used in future intervention studies to control serum lipids and thus reduce the risk of CVD.

The DDKQ scores of females, participants with a higher educational level, those with greater income, and urban dwellers were higher than the scores of those without these characteristics. Currently, no specific instrument exists to measure dyslipidemia dietary knowledge. However, Bonaccio et al. [31] found that people in the highest quartile of nutrition knowledge were predominately women, had a higher educational level, and had higher income. Chen et al. [32] found that rural participants had significantly lower nutritional knowledge and self-efficacy. These are similar to the results of the current study. However, there were no significant differences according to the presence or absence of dyslipidemia, or age. Interestingly, scores of individuals with abnormal blood lipids known by laboratory tests did not differ from scores of other individuals, indicating the lack of knowledge of the former, who may not have been aware of the hazards dyslipidemia presents to their own health. Wang et al. [33] showed that only 28.5% of Chinese residents are aware of dyslipidemia. However, based on data from the World Health Organization Multinational Monitoring of Trends and Determinants in Cardiovascular Disease project, the average hypercholesterolemia awareness is only 36% (ranging from 3% [0%] to 62% [65%] in men [women]) [34]. Therefore, it is particularly important to improve awareness of dyslipidemia. The current results also show that exceptional knowledge of the dyslipidemia diets is unrelated to age. In addition, Zhang et al. [35] show that dyslipidemia currently occurs in every age group, with increasingly many young individuals experiencing the condition in China. Therefore, it is very important to strengthen health education related to blood lipids.

The DDKQ consists of multiple-choice questions (MCQs). Written tests can have various formats, but the most optimal is MCQs because multiple-choice question tests can assess a large proportion of the relevant knowledge or curriculum, can be used repeatedly, possess high reliability, and reduce response and scoring times [36, 37]. When well-constructed, the central asset of MCQs is their ability to comprehensively assess the test taker's knowledge [37, 38]. It is precisely because the DDKQ uses multiple choice questions that a new evaluation tool could be employed, namely, non-functioning distractors (NFDs). NFDs affect difficulty and discrimination; in general, NFDs will decrease item difficulty. For example, the index of difficulty for item 7 in Step 3 was 0.86, and after adjustment, it became 0.82 in Step 4. In Step 3 the item had 3 NFDs. After modification, the distractor items became functional, thus improving the questionnaire.

The DDKQ's mean item difficulty was 0.55, indicating moderate difficulty; discrimination was also good. The DDKQ, consisting of 25 items of varying difficulty levels, was used to evaluate individuals' dyslipidemia dietary knowledge in this study. However, for two of the intake-related items, namely, “How many grams of meat (meat, poultry) should an adult ingest daily?” and “In dyslipidemia, the daily recommended intake of cholesterol is less than?”, only 15% and 32% of individuals knew the specific appropriate intake, respectively (i.e., item difficulty levels were 0.15 and 0.32, respectively). The results indicate that the majority of people lack this component of knowledge about recommended intake. Although most people do not measure their meat to the gram or their cholesterol intake to the milligram, they should know how much recommended daily intake is, and how to estimate the gram, then they could translate the knowledge into the formation of good diet habits. How to transform those abstract knowledge into specific and operable behavior is the exploring direction of our future studies.

The reliability of the DDKQ was analyzed in terms of its internal consistency, as assessed by the Kuder-Richardson 20. Since dyslipidemia dietary knowledge is a multidimensional construct (including fat knowledge, cholesterol knowledge, dietary fiber knowledge, phytosterol knowledge, total energy knowledge, and healthy lifestyle knowledge), the Kuder-Richardson 20 is not necessarily critically important in this case [39]. Although the current version of the dyslipidemia dietary knowledge questionnaire had less than the stated acceptable level of 0.70 for internal consistency, some psychometricians deem values between 0.65 and 0.70 as “minimally acceptable” [40]. In addition, a previous study have concluded that KR20 formula between 0.5 and 0.7 was considered the minimum acceptable for internal consistency [41].

Regarding construct validity, the scores of a randomly selected 46 outs of 496 investigators were significantly lower than those of 46 medical workers, indicating that the questionnaire is capable of discriminating between two populations with different degrees of knowledge. The medical workers were primarily from university nutrition departments, and some were based in departments of cardiology or endocrinology. Such backgrounds include health-related education. No comparison was carried out between this questionnaire and any other instrument because there is no general diet knowledge questionnaires reported in the Chinese literature. In addition, considering the difference of diet culture, the current questionnaire could not have been compared to foreign questionnaire.

This questionnaire was developed not only to characterize high or low scores, but also to clarify the nature of any deficits in knowledge (i.e., pertaining to fat, cholesterol, dietary fiber, total energy, healthy lifestyle). This allows for targeted health education guidance. Test scores can reflect the test taker's mastery of knowledge. However, the process of completing the questionnaire is a process of self-reflection: questionnaire completion may make the test taker aware of whether he or she lacks knowledge or understanding. Scores may also be obtained before and after an intervention, to evaluate its effectiveness.

There were some limitations to our study. First, the DDKQ is suitable for Chinese food culture, with reference to Chinese dietary guidelines. Due to differences in Asian and Western food cultures, the questionnaire is only suitable for areas such as China and Singapore. However, our questionnaire design could provide the reference for other countries to develop the suitable diet knowledge questionnaire, and to make the tool more widely used, because the incidence of dyslipidemia is high in the world. Furthermore, most of the participants (77.12%) came from urban areas; therefore, the questionnaire should be tested on those from rural areas or remote regions of China. Finally, this survey has not been tested to see whether knowledge scores are associated with diet, or with successful diet change; this was beyond the scope of the current research, and that it would be an important step in further validation to help clinicians with education or assessment.

The questionnaire is a knowledge scale and might facilitate the design of food pictures and models to assist future investigations, to enrich the knowledge of different kinds of food, and to advocate for healthier ways of life.

## 6. Conclusions

This study developed a practical 25-item instrument to evaluate knowledge of dyslipidemia diets. The DDKQ provided scores with good reliability and validity. The findings also highlighted the importance of health education regarding dietary management of CVD, as many respondents lacked the essential knowledge to combat CVD. Adequate knowledge of diet and healthy lifestyle may be effective against the current high incidence of CVD, and its social and economic burden.

## Figures and Tables

**Table 1:** Demographic characteristics of participants included in Step 3.

Characteristic	N	Proportion (%)
*Gender*		
Male	156	46.02
Female	183	53.98
*Age *		
18–44	143	42.18
45–59	102	30.09
≥60	94	27.73
*Dyslipidemia*		
Yes	149	43.95
No	190	56.05

**Table 2:** Demographic characteristics of participants included in Step 4.

Characteristic	N (%)	DDKQ score		*p*
		Mean	SD	
*Gender*				<0.05
Male	249(45.94)	13.12	3.89	
Female	293 (54.06)	14.09	3.66	
*Age *				0.245
18–44	189 (34.87)	13.61	3.94	
45–59	176 (32.47)	13.31	3.59	
≥60	177 (32.66)	13.98	3.82	
*Dyslipidemia*				0.461
Yes	268 (49.45)	13.51	3.78	
No	274 (50.55)	13.75	3.81	
*Home address*				<0.05
Rural	124 (22.88)	12.15	3.40	
Urban	418 (77.12)	14.07	3.74	
*Educational level*				<0.05
Elementary/junior middle school	184 (33.95)	12.28	3.52	
Senior middle school/junior college	151 (27.86)	13.74	3.63	
Junior college/university	182 (33.58)	14.79	3.69	
Graduate school or above	25 (4.61)	14.56	4.37	
*Average monthly family income*				<0.05
<1000 RMB	39 (7.20)	12.38	3.92	
1000–2000 RMB	131 (24.2)	12.51	3.81	
2000–3000 RMB	166 (30.6)	13.29	3.77	
3000–4000 RMB	109 (20.1)	14.68	3.29	
≥4000 RMB	97 (17.9)	15.06	3.58	

RMB, Ren Min Bi: Chinese currency.

**Table 3:** The CVI, P, and D of each item of the final DDKQ.

Part	Item	CVI	P	D
Part1: Knowledge of fats	1. Fat mainly exists in the following foods	1.00	0.82	0.33
	2. In dyslipidemia, the preferred meat is	0.93	0.60	0.55
	3. Adult daily calories from fat does not exceed	1.00	0.41	0.28
	4. In dyslipidemia, which of the following types of pork are the best choice	0.96	0.71	0.45
	5. How many grams of meat (meat, poultry) should an adult ingest daily	0.85	0.15	0.21
	6. The adult daily cooking oil volume should not exceed (1 spoon ≈10g)	1.00	0.33	0.29
	7. In dyslipidemia, preferred cooking methods are	0.96	0.82	0.35
	8. Adults should limit their intake of fatty acids	0.89	0.39	0.30
	9. Eating a lot of trans fatty acids will accelerate atherosclerosis and lead to cardiovascular disease. In the following foods, trans fatty acid content is minimal	0.96	0.66	0.46
	10. The saturated fatty acid content of which oil is the highest?	0.78	0.74	0.28

Part 2: Knowledge of cholesterol	11. The food that contains the most cholesterol is	1.00	0.79	0.35
	12. In dyslipidemia, the daily recommended intake of cholesterol is less than	0.96	0.32	0.24
	13. Foods that lower cholesterol are	0.93	0.78	0.36

Part 3: Knowledge of dietary fiber	14. Dietary fiber mainly exists in the following types of food	0.96	0.83	0.32
	15. The dietary fiber content of which of these foods is the highest	0.89	0.43	0.22
	16. Which of these staple foods has the highest fiber content?	0.89	0.46	0.58
	17. Dietary fiber content is minimal in porridge	0.89	0.44	0.44

Part 4: Total energy knowledge	18. Which of the following is not a high-calorie, high-sugar food	0.93	0.77	0.38
	19. The foods that contain the most carbohydrate are	0.96	0.44	0.47
	20. A suitable intake of adult daily staple foods (valley potato and beans) is	0.93	0.36	0.34
	21. The following foods are preferred in dyslipidemia	1.00	0.75	0.46

Part 5: Healthy lifestyle knowledge	22. Three meals a day is a reasonable food intake distribution method	0.93	0.33	0.22
	23. The proper amount of vegetable intake per day is	0.96	0.46	0.40
	24. The appropriate intake of fruit per day for adults is	0.96	0.50	0.23
	25. The recommended intake of salt in adults is less than	0.93	0.51	0.55

Average value		0.94	0.55	0.36

CVI, content validity index, CVI ≥0.78 was considered excellent, otherwise the item was deleted. P, difficulty (of a test item), P ≥ 0.9 or P ≤0.1 were considered too easy or too difficult and were deleted. D, discrimination index (of a test item), < 0.20 denoted an item with low discriminatory power that was deleted [26].

## Data Availability

The data used to support the findings of this study are included within the article.
